# Rationale and design of the iCORONARY trial: improving the cost-effectiveness of coronary artery disease diagnosis

**DOI:** 10.1007/s12471-023-01758-3

**Published:** 2023-01-31

**Authors:** J. Peper, L. M. Becker, T. A. Bruning, R. P. J. Budde, W. G. van Dockum, G. W. J. Frederix, J. Habets, J. P. S. Henriques, P. Houthuizen, F. A. A. Mohamed Hoesein, R. N. Planken, M. Voskuil, M. L. Bots, T. Leiner, M. J. Swaans

**Affiliations:** 1 Department of Cardiology, St. Antonius Hospital, Nieuwegein, The Netherlands; 2 Department of Radiology, University Medical Centre Utrecht, Utrecht, The Netherlands; 3 Department of Cardiology, Maasstad Hospital, Rotterdam, The Netherlands; 4 Department of Radiology, Erasmus Medical Centre, Rotterdam, The Netherlands; 5 Department of Public Health, Healthcare Innovation and Evaluation and Medical Humanities, University Medical Centre Utrecht, Utrecht, The Netherlands; 6 Department of Radiology, Radboud University Medical Centre, Nijmegen, The Netherlands; 7 Department of Cardiology, Amsterdam University Medical Centre, Amsterdam, The Netherlands; 8 Department of Cardiology, Catharina Hospital, Eindhoven, The Netherlands; 9 Department of Radiology, Amsterdam University Medical Centre, Amsterdam, The Netherlands; 10 Department of Cardiology, University Medical Centre Utrecht, Utrecht, The Netherlands; 11 Julius Centre for Health Sciences and Primary Care, University Medical Centre Utrecht, Utrecht University, Utrecht, The Netherlands; 12 Department of Radiology, Mayo Clinic Hospital, Rochester, United States of America

**Keywords:** Coronary artery disease, Coronary computed tomography angiography, Fractional flow reserve, Computed-tomography-derived fractional flow reserve, Quantitative flow ratio

## Abstract

**Background:**

In patients with stable coronary artery disease (CAD), revascularisation decisions are based mainly on the visual grading of the severity of coronary stenosis on invasive coronary angiography (ICA). However, invasive fractional flow reserve (FFR) is the current standard to determine the haemodynamic significance of coronary stenosis. Non-invasive and less-invasive imaging techniques such as computed-tomography-derived FFR (FFR-CT) and angiography-derived FFR (QFR) combine both anatomical and functional information in complex algorithms to calculate FFR.

**Trial design:**

The iCORONARY trial is a prospective, multicentre, non-inferiority randomised controlled trial (RCT) with a blinded endpoint evaluation. It investigates the costs, effects and outcomes of different diagnostic strategies to evaluate the presence of CAD and the need for revascularisation in patients with stable angina pectoris who undergo coronary computed tomography angiography. Those with a Coronary Artery Disease—Reporting and Data System (CAD-RADS) score between 0–2 and 5 will be included in a prospective registry, whereas patients with CAD-RADS 3 or 4A will be enrolled in the RCT. The RCT consists of three randomised groups: (1) FFR-CT-guided strategy, (2) QFR-guided strategy or (3) standard of care including ICA and invasive pressure measurements for all intermediate stenoses. The primary endpoint will be the occurrence of major adverse cardiac events (death, myocardial infarction and repeat revascularisation) at 1 year. Clinicaltrials.gov-identifier: NCT04939207.

**Conclusion:**

The iCORONARY trial will assess whether a strategy of FFR-CT or QFR is non-inferior to invasive angiography to guide the need for revascularisation in patients with stable CAD. Non-inferiority to the standard of care implies that these techniques are attractive, less-invasive alternatives to current diagnostic pathways.

## Introduction and rationale

Invasive coronary angiography (ICA) and invasive pressure measurements such as fractional flow reserve (FFR) are used as the reference standard for the diagnosis of the haemodynamic significance of coronary stenosis. Both ICA and invasive pressure measurements are considered low-risk invasive procedures. If complications occur, these are generally mild, such as bleeding or haematoma at the access location, which happens in approximately 5% of the patients. More serious complications as compartment syndrome, dissection of vessels, myocardial infarction, stroke and cardiac arrhythmias including ventricular fibrillation are rare. Event rates vary between approximately 1/1000 and 1/100,000 procedures, but since ICA and invasive pressure measurements are frequently performed, absolute numbers of serious complications are significant [1]. Besides the additional risks, invasive pressure measurements are an expensive strategy with costs amounting to €1000 per patient in the Netherlands (excluding the costs of catheterisation) [2].

ICA and invasive pressure measurements are not useful if revascularisation is not feasible and expected. Although the rates vary per hospital, approximately 50% of all patients currently referred for invasive tests in the Netherlands do not have a significant lesion, and do not need revascularisation [2]. While clinical evaluation, non-invasive imaging and stress testing are needed for risk stratification in patients with suspected coronary artery disease (CAD), diagnostic over-testing needs to be avoided. The lack of consensus in the current guidelines about the optimal diagnostic pathway for patients with suspected CAD results in major differences in strategies between hospitals. A budget impact analysis performed by the Dutch National Healthcare Institute shows a potential decrease in costs of €177 million per year with improvements in the diagnostic strategy for stable CAD. In addition, favourable effects on the health of patients are expected because of averted side-effects of unnecessary invasive testing [2]. Non-invasive imaging techniques improve the diagnostic process, but the exposure to radiation and/or contrast agent can lead to complications, which increase costs and negatively affect the quality of life. Moreover, most of the diagnostic tests focus either on anatomical or functional information.

New non-invasive and less-invasive imaging techniques, for example computed-tomography-derived FFR (FFR-CT) and angiography-derived FFR [quantitative flow ratio (QFR)], combine both anatomical and functional information. The PLATFORM and FORECAST studies showed conflicting results regarding differences in costs between an FFR-CT-guided strategy and usual care and no differences in clinical outcomes, but the use of ICA decreased [3]. Both FFR-CT and QFR are expected to be cheaper than invasive pressure measurements. Costs amount to approximately €1000 per patient for FFR-CT [excluding the costs for coronary computed tomography angiography (CCTA)] and approximately €500 for QFR (excluding the costs for ICA). We hypothesise that FFR-CT and QFR are more cost-effective in the Dutch healthcare system in comparison to invasive pressure measurements by lowering the percentage of patients referred for invasive pressure measurements. FFR-CT also increases patient comfort and could lead to a lower rate of complications, which adds to its cost-effectiveness. However, evidence is conflicting or lacking and requires more head-to-head cost-effectiveness studies of alternatives to invasive pressure measurements.

The aim of the iCORONARY trial is to determine whether non-invasive or minimal-invasive imaging techniques such as FFR-CT and QFR are a cost-effective and safe alternative to invasive pressure measurements when deciding on the indication for revascularisation in patients with suspected CAD in terms of subsequent major adverse cardiac events (MACE).

## Methods

### Trial design

**Fig. 1:**
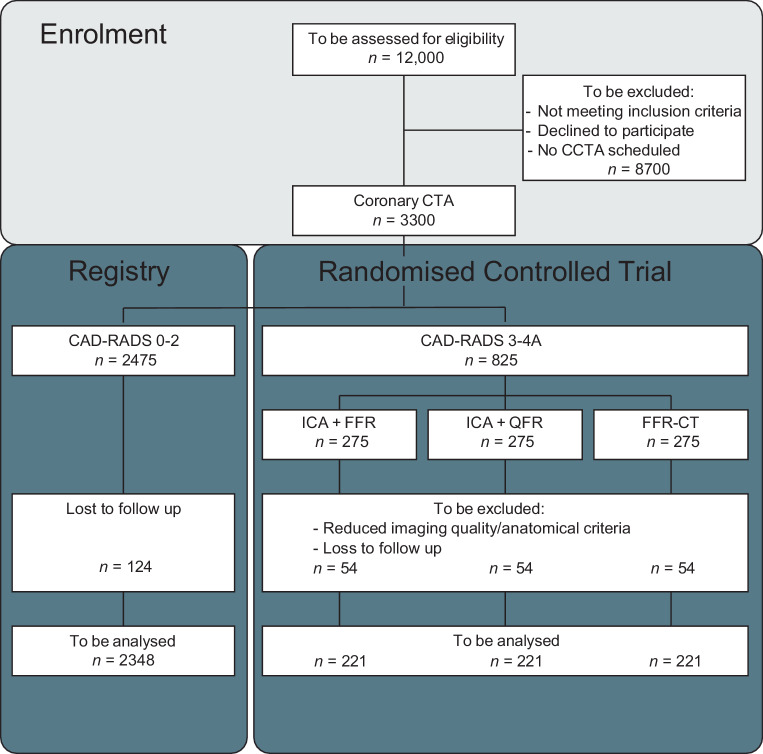
Study flow chart. Patients are considered “lost to follow-up” if they retract their informed consent at any point during the study. In this case, data collected so far can still be used. Information regarding death of a subject will be obtained via medical records, the patient’s general practitioner and Statistics Netherlands. *CAD-RADS* Coronary Artery Disease—Reporting and Data System, *CCTA* coronary computed tomography angiography, *FFR* fractional flow reserve, *FFR-CT* computed tomography derived fractional flow reserve, *ICA* invasive coronary angiography, *QFR* quantitative flow ratio

This study is a prospective, multicentre, non-inferiority randomised controlled trial (RCT) with an open, blinded endpoint evaluation (PROBE design) investigating the costs, effects and outcomes of different diagnostic strategies to establish the presence or absence of flow-limiting coronary artery stenoses in need of revascularisation according to the guidelines. The study design is pragmatic and closely follows currently used diagnostic pathways and tests in clinical practice (Fig. 1).

### Patient selection

This study concerns patients referred to a cardiologist with chest pain of suspected coronary origin. Only patients who had no prior coronary interventions are eligible to participate. Detailed inclusion and exclusion criteria are listed in Tab. 1. All eligible patients will undergo CCTA. CCTA images will be assessed using the standardised Coronary Artery Disease—Reporting and Data System (CAD-RADS) [4]. The CAD-RADS score distinguishes patients with absence of CAD (CAD-RADS 0) and mild, non-obstructive stenosis (CAD-RADS 1 and 2) from patients with moderate or higher degrees of stenosis (CAD-RADS 3–5). After giving signed informed consent, participants are recruited based on their CAD-RADS result into either the registry part or the randomised part of the study:

- Patients with CAD-RADS between 0–2 and 5 are included in a prospective multicentre registry in which we will assess outcomes at 12 months after CCTA. Follow-up of these patients ensures we obtain a “real-world” estimate of the ability of CCTA to adequately triage patients with no or low CAD disease burden from patients with intermediate to severe CAD disease burden as practiced in the Netherlands.
- Patients with CAD-RADS 3 or 4A in at least one vessel are included in the RCT and randomised to: (1) a usual care arm, (2) an FFR-CT-guided revascularisation arm, and (3) a QFR arm.

**Table 1:** Inclusion and exclusion criteria, which are similar for both the registry and the randomised controlled trial (RCT). Patients with a Coronary Artery Disease—Reporting and Data System (CAD-RADS) score of 3 or 4A in at least one vessel are included in the RCT, whereas patients with CAD-RADS 0–2 or 5 are eligible for the registry

Inclusion criteria	Exclusion criteria
The subject is ≥ 18 years of age	The subject is suffering from unstable angina pectoris
The subject is willing and able to provide informed consent and to adhere to study rules and regulations as well as follow-up requirements	The subject is suffering from decompensated congestive cardiac failure
	The subject is suffering from a known non-ischaemic cardiomyopathy
There is a clinical suspicion of (recurrent) angina pectoris or an equivalent symptom and suspected coronary artery disease, based on symptoms and signs, history, clinical examination and baseline diagnostic testing (e.g. ECG recording and laboratory tests) as described in the 2019 ESC guideline on chronic coronary syndromes	The subject has a history of percutaneous coronary intervention or coronary artery bypass grafting
	The subject has had pacemaker or internal defibrillator leads implanted
	The subject has a prosthetic heart valve
	There is a severe language barrier
	The subject is participating in any other clinical trial that interferes with the current study
The subject has had ≥ 64 multidetector row coronary CTA or will undergo coronary CTA as part of usual care deemed necessary by the treating physician with ≥ 64 multidetector row coronary CTA	The subject’s clinical condition prohibits subsequent interventional therapy as indicated by the results of the imaging procedures
	The subject is or might be pregnant
	The subject does not comply or is not able to comply with the imaging guidelines for the performance and acquisition of coronary CTA as defined by the SCCT

*CTA* computed tomography angiography, *ECG* electrocardiogram, *ESC* European Society of Cardiology, *SCCT* Society of Cardiovascular Computed Tomography

### Primary outcome

The primary clinical outcome for both the registry and the RCT is the occurrence of MACE within 12 months of follow-up. This composite endpoint includes all-cause mortality, aborted sudden cardiac death, myocardial infarction and unplanned hospitalisation for chest pain leading to urgent revascularisation (Tab. 2).

**Table 2:** Definitions of primary and secondary endpoints, which are similar for both the registry and the randomised controlled trial

Endpoint	Definition
*Primary endpoint and components*	
Major adverse cardiac events	Composite of all-cause mortality, aborted sudden cardiac death, myocardial infarction and unplanned (acute) hospitalisation leading to urgent revascularisation
All-cause mortality	Death of a subject during the study, defined as 12 months after the inclusion of the final subject, regardless of the cause or circumstances of death
Aborted sudden cardiac death	Successful reversal of unexpected circulatory arrest, with the arrest occurring within 1 h of onset or worsening of acute symptoms, by resuscitation manoeuvres (i.e. chest compressions, cardiac defibrillation), resulting in sustained return of spontaneous circulation or sustained extracorporeal circulatory support for at least 20 min [16, 17]
Myocardial infarction	Acute myocardial infarction type 1–3 and procedure-related myocardial infarction type 4 and 5 as defined by the fourth universal definition of myocardial infarction [18]
Unplanned hospitalisation leading to urgent revascularisation	Unplanned hospitalisation due to chest pain or other symptoms suspected to be caused by myocardial ischaemia, but not meeting the criteria for myocardial infarction as defined by the fourth universal definition of myocardial infarction, resulting in urgent coronary revascularisation
*Secondary endpoints*	
Unstable angina	Defined by the ESC as myocardial ischaemia at rest or on minimal exertion in the absence of acute cardiomyocyte injury/necrosis [19]
Non-fatal stroke	Survival of at least 28 days after the onset of stroke, defined as a focal (or at times global) neurological impairment of sudden onset of presumed vascular origin, lasting more than 24 h (or resolved by treatment but expected to have lasted more than 24 h in the absence of treatment) [20]
Avoided ICA procedures	The number of ICA procedures that were not performed due to the availability of FFR-CT, but that would have been indicated based on the results of non-invasive diagnostic tests if FFR-CT had not been available
Avoided FFR measurements	The number of FFR measurements that were not performed due to the availability of QFR, but that would have been indicated based on the anatomical stenosis severity visible on angiographic images if QFR had not been available

*ESC* European Society of Cardiology, *FFR* fractional flow reserve, *FFR-CT* computed tomography derived fractional flow reserve, *ICA* invasive coronary angiography, *QFR* quantitative flow ratio

### Secondary outcome

The secondary outcomes include the individual components of the primary outcome, a composite endpoint of unstable angina and other hospitalisations for cardiac reasons and angina frequency and stability, physical limitations, treatment satisfaction and quality of life. In the RCT the cost-effectiveness, the proportion of ICA procedures not performed owing to the availability of FFR-CT and the proportion of invasive FFR measurements avoided by the use of QFR will be assessed.

### Study procedures

#### Registry

The registry includes patients with CAD-RADS 0–2 and 5. These patients will be treated as determined by the referring physician team and includes guideline conformant optimal medical therapy (OMT). After inclusion, patients in the usual care arm will not undergo any additional invasive tests or procedures.

#### Randomisation arm FFR-CT

The CCTA of patients randomised to the FFR-CT arm will be analysed by HeartFlow FFR-CT (HeartFlow, Mountain View, CA, USA). Participating hospitals will follow local CCTA scanning protocols consistent with quality standards as defined by the Society of Cardiovascular Computed Tomography [5]. Prior to the FFR-CT analyses, the quality of CT images will be evaluated by the local radiologist and quantitatively scored for all vessel segments ≥ 2 mm in diameter to select cases appropriate for FFR-CT analysis.

FFR-CT analyses will be performed on the resting CCTA images using the HeartFlow Core Lab. It is expected that the CCTA of 10–15% of the enrolled subjects will be of acceptable quality [6, 7]. Haemodynamically significant CAD will be defined as FFR-CT ≤ 0.80 in any segment distal from a coronary stenosis with a reference size of ≥ 2.0 mm. If haemodynamically significant CAD is present in a segment suitable for revascularisation, these patients will be referred for ICA and undergo revascularisation. Patients with FFR-CT values > 0.80 in all segments will not be referred for ICA and will receive OMT.

#### Randomisation arm QFR

Patients randomised to the QFR arm will undergo ICA to visualise the coronary arteries. The diagnostic ICA will be performed by certified interventional cardiologists using imaging standards defined by the ACC/AHA Task Force on Practice Guidelines and the Society for Cardiac Angiography and Interventions. QFR will be estimated in the catheterisation laboratory immediately after injection of contrast if there is at least one lesion with a diameter stenosis between 30% and 90% in a vessel with reference size ≥ 2.0 mm. QFR will be calculated using the QAngio XA 3D/QFR analytical software solution (Medis Medical Imaging Systems, Leiden, The Netherlands). To obtain a QFR measurement, two angiographic views of the vessel of interest at least 25° apart are obtained on the least foreshortening of the stenosis and a minimum overlap between the main vessels and side branches. Haemodynamically significant CAD is defined as QFR ≤ 0.80. QFR will be performed on-site by QFR-certified observers. It is expected that in 1–6% of enrolled patients the angiographic views will not fulfil the above criteria and that QFR can therefore not be performed [8–10].

#### Randomisation arm standard of care

Patients randomised to the usual care arm will, in cases with an intermediate severity stenosis (50–90% diameter stenosis) that can be safely measured by invasive means, undergo ICA and invasive pressure measurements in accordance with the ESC guidelines. The management strategy (percutaneous coronary intervention or coronary artery bypass graft or OMT) will be determined based on this information. Haemodynamically significant CAD is defined as FFR ≤ 0.80 and instantaneous wave-free ratio and resting full-cycle ratio ≤ 0.89.

#### Study endpoints and questionnaires

Clinical endpoints, mortality and MACE will be obtained from the electronic patient records and other data sources (general practitioner, national death registry and participants themselves) until the end of the study (at least 12 months’ follow-up for each participant, maximum follow-up 30 months). A blinded clinical endpoint committee will adjudicate all endpoints.

Symptoms of angina are recorded by use of the Seattle Angina Pectoris Questionnaire and mental status is assessed using PHQ‑9. Medical costs and productivity losses are recorded using the iMTA Medical Consumption Questionnaire and iMTA Productivity Cost Questionnaire (iMTA, Rotterdam, The Netherlands). Health-related quality of life, as measured by the Patient-Reported Outcome Measurement Information System (PROMIS-10), including the score on the European Quality of Life 5 Dimensions 5 Levels questionnaire (EQ-5D-5L; EuroQol, Rotterdam, The Netherlands), will be established at baseline and 1, 3, 6 and 12 months after inclusion. In addition, patients will be asked to complete a test rating scale for discomfort and satisfaction with the testing procedure. Patients who receive an invasive pressure measurement after the FFR-CT or QFR test will be asked to compare the tests directly.

### Statistical considerations

All analyses will be done according to the intention-to-treat principle defined as all subjects randomised into the trial. Treatment classification will be based on the result of the randomly allocated diagnostic test. The per-protocol population will be defined as all subjects randomised into the trial receiving their assigned randomised treatment.

#### Sample size

The sample size calculation is based on the expected percentage of patients with the primary composite endpoint of MACE at 12 months. A 3.7% incidence in the FFR-guided arm and a non-inferiority margin of 5% are assumed based on the results of the MR-INFORM trial [11]. With these assumptions, a sample size of 221 patients in each arm is estimated to determine non-inferiority (one-sided level of significance of 0.025) and to provide the trial with at least 80% power. Based on local experience approximately 25% of the patients evaluated by CCTA have a CAD-RADS score of 3 or 4A. The pertinent literature suggests that a maximum of 15% of the patients are not suitable for FFR-CT or QFR analyses [8, 9, 12–14]. Allowing for an exclusion percentage of 15% and a dropout rate caused by loss to follow-up of 5%, a total sample size of 3300 patients is required. It should be noted that 75% of these patients are asked for follow-up only (CAD-RADS 0–2), and 25% (*n* = 825) will be randomised to the different techniques to measure FFR.

#### Data analyses

The primary endpoint will be assessed in a non-inferiority analysis of the FFR-CT-guided group and the QFR-guided group compared to the conventional strategy-guided group. The null hypothesis is that the MACE incidence rates of both FFR-CT and QFR are equal to or lower than the incidence rates of the usual care arm within the non-inferiority margin of 5%. If non-inferiority is confirmed, superiority will be assessed. Differences in the primary outcome will be assessed graphically using Kaplan-Meier curves and tested with the log-rank test as recommended for an analytical approach to a three-arm non-inferiority trial [15]. In addition, analysis considering crossovers will be performed. No formal interim analysis for efficacy is planned for this study.

### Ethical considerations

This study will be conducted according to the principles of the Declaration of Helsinki and in accordance with the most recent European Good Clinical Practice rules and the ISO 14155:2020. Major adverse events will be reported to the Data Safety Monitoring Board (DSMB) and the accredited medical research ethics committee. The DSMB reviews adverse event data, other safety data, quality and completeness of study data, and enrolment data to ensure proper trial conduct.

### Funding, trial registration and time line

The iCORONARY trial is registered on ClinicalTrials.gov (NCT04939207). The trial was initiated as a collaboration between St Antonius Hospital and University Medical Centre Utrecht, and more than eight Dutch centres are anticipated to include patients. The iCORONARY trial is funded by the Dutch Organization for Health Research and Development (ZonMW) and the health insurance companies in the Netherlands (grant number: 852002131), none of which are involved in trial design and processes.

Initial recruitment at the St Antonius Hospital began in March 2022. The trial will continue until 3300 patients are included and followed for 12 months.

## Summary

The iCORONARY trial is a multicentre, prospective, randomised controlled, non-inferiority trial that assesses whether a non-invasive or less-invasive strategy for FFR-CT or QFR is non-inferior to invasive angiography and FFR measurements in guiding the need for revascularisation in patients with stable CAD. Non-inferiority of FFR-CT and/or QFR to the current standard of care would establish those procedures as an attractive, cost-effective, non-invasive or less invasive alternative to current diagnostic pathways. The results of the iCORONARY trial might contribute to the guidelines on diagnosis and management of stable CAD.
